# Improving retrospective ARDS case-finding using a simple 72-h physiologic persistence rule

**DOI:** 10.1186/s40635-026-00901-9

**Published:** 2026-04-30

**Authors:** Dominic C. Marshall, Brijesh V. Patel, Anthony C. Gordon, David B. Antcliffe, Sonali Parbhoo, Matthieu Komorowski

**Affiliations:** 1 Division of Anaesthetics, Pain Medicine and Intensive Care, Department of Surgery and Cancer, Imperial College London, London, UK; 2 Cleveland Clinic London, London, UK; 3 School of Electrical and Electronic Engineering, Imperial College London, London, UK; 4 Charing Cross Hospital, London, UK

**Keywords:** Acute respiratory distress syndrome, Identification, Phenotyping, Case-finding, Persistence

## Abstract

**Background:**

Retrospective studies frequently use single-time-point Berlin physiologic criteria (PaO_2_/FiO_2_ < 300 mm Hg plus positive end-expiratory pressure ≥ 5 cm H_2_O) to identify acute respiratory distress syndrome (ARDS). However, transient hypoxaemia is common among critically ill patients and often does not represent ARDS. Using these screens may overestimate ARDS prevalence and yield a cohort inconsistent with those seen in clinical trials.

We sought to determine whether a 72-h persistence criterion for hypoxaemia improves the accuracy of ARDS case identification and evaluate the additional case-finding value of radiology keyword searches and ICD-codes.

**Methods:**

We conducted a retrospective cohort study using the MIMIC-IV database (2008–2019) for derivation and a UK ICU dataset (Imperial College Healthcare National Health Service Trust, 2009–2024) for external validation. All patients meeting Berlin physiologic criteria for at least 72 h were identified. From MIMIC-IV, we randomly selected 2000 patients who met 72-h persistence criteria for expert adjudication based on detailed review of clinical notes, imaging, and echocardiography, classifying them as ARDS, non-ARDS acute hypoxaemic respiratory failure, or possible ARDS. Sensitivity analyses with shorter durations (≥ 24 and ≥ 48 h) were performed. Diagnostic performance of radiology keyword searches and ARDS-specific ICD-9/10 codes were compared to expert adjudication.

**Results:**

Of 18,621 patients who ever met physiologic criteria, 3940 met the 72-h persistence threshold. In a random sample of 2000 from this 72-h MIMIC-IV cohort, expert adjudication identified ARDS in 49.7% (95% CI, 48–52%); in the external UK validation cohort, 56% (95% CI, 46–66%) were adjudicated as ARDS. ARDS prevalence significantly declined with shorter persistence requirements: 21% after 48 h, 8% after 24 h, and 6% with single isolated measurements. Within the 72-h persistence criterion enriched sample, the highest performing radiology keyword search set provided limited sensitivity (49%) and moderate specificity (76%), whereas ICD codes had higher sensitivity (76%) but low specificity (47%).

**Conclusions:**

Berlin physiologic criteria alone were inadequate for retrospective ARDS identification. A ≥ 72-h persistence rule improved cohort enrichment but did not define ARDS, with substantial residual misclassification remaining after physiologic screening. Persistence should therefore be viewed as a pragmatic enrichment strategy rather than a definitive retrospective ARDS label.

**Supplementary Information:**

The online version contains supplementary material available at 10.1186/s40635-026-00901-9.

## Introduction

Acute respiratory distress syndrome (ARDS) is a clinical syndrome of non-cardiogenic pulmonary oedema that produces severe hypoxaemia and bilateral opacities on imaging and remains a leading cause of mortality on the intensive care unit (ICU). Despite advances in lung-protective ventilation, prone positioning, and extracorporeal membrane oxygenation, few new therapies have changed practice. Current efforts emphasise precision-medicine approaches that account for clinical, biologic, and radiologic phenotypes [1–4].

Large public ICU databases such as MIMIC-IV [5], the eICU-Collaborative Research Database [6], and the Amsterdam UMC database [7] now allow investigators to study tens of thousands of ventilated patients at minimal cost and have already been used to explore the new 2023 Global Definition of ARDS, derive machine-learning “sniffers” and describe outcome-relevant phenotypes [8–10]. However, retrospective case-finding is vulnerable to major misclassification. ARDS is under-recognised and under-coded [11–13], with retrospective chart review requiring high-granularity physiology, imaging and clinical context.

Many studies rely solely on the physiological component of the Berlin criteria (PaO_2_/FiO_2_ (P/F) < 300 mm Hg + positive end expiratory pressure [PEEP] ≥ 5 cm H_2_O), sometimes in combination with keyword searches of chest radiograph reports or ICD codes [11, 14–19]. These approaches may be easy to implement; however, exclusion of cardiogenic pulmonary oedema, fluid overload or transient hypoxaemia is important for accurate classification. Further, definitions lack an explicit temporal component, so transient hypoxaemia can satisfy physiologic thresholds. At the bedside, clinicians typically require sustained hypoxaemia before diagnosing ARDS, and several trials introduced stabilisation windows (e.g. 12–24 h before enrolment) [3], underscoring the importance of persistence. This gap raises the possibility that many retrospective cohorts differ from those enrolled in ARDS trials.

We therefore conducted a pilot review of 100 mechanically ventilated patients using a single 24-h physiological Berlin-criteria screen and found that only 6% satisfied the full case definition. As open-lung biopsy is now rarely undertaken, clinicians must rely on syndromic criteria; nevertheless, diffuse alveolar damage (DAD) remains the likely common histopathological pathway, and autopsy series suggest it is uncommon when hypoxaemia lasts < 72 h but present in 62% of patients whose impairment persists ≥ 72 h [20]. These observations support the use of a persistence-based (≥ 72 h) physiologic enrichment strategy, with our phenotype of interest being clinically adjudicated ARDS, which aligns with the syndromic definitions used at the bedside and in research.

Our primary objective was to determine, in a large ICU database with external validation, what proportion of patients who meet commonly used physiological screening criteria are consistent with clinical ARDS and whether 72 h of hypoxaemia improves accuracy. The secondary objective was to assess the utility of additional enrichment strategies to improve the accuracy of retrospective case-finding.

## Methods

### Data sources and design

We conducted a retrospective cohort study using two ICU databases. The derivation dataset was MIMIC-IV v1.0 (Beth Israel Deaconess Medical Center, 2008–2019). Demographic, physiologic, laboratory, ventilator, and narrative data were extracted using SQL on a Google Cloud mirror. Chest-radiograph reports (2011–2016) were obtained from the linked MIMIC-CXR resource. The external validation dataset comprised ICU admissions (2009–2024) from three hospitals within Imperial College Healthcare National Health Service Trust (ICHT) using the Philips IntelliSpace Critical Care & Anaesthesia system. Clinical notes, imaging, and free-text reports were available for manual review. Institutional approvals and data-use agreements were obtained as required.

The study proceeded in sequential phases: (1) pilot sampling and time-based threshold selection; (2) cohort identification with a 72-h physiologic screen and expert review in MIMIC-IV; (3) assessment of alternative enrichment strategies; (4) sensitivity cohorts using 24-h and 48-h screens; and (5) external validation in ICHT.

### Data processing

Physiologic, ventilator, and blood gas measurements were harmonised to ensure temporally contemporaneous P/F and PEEP values. For each 24-h window, we used the mean P/F value contemporaneous with PEEP ≥ 5 cm H_2_O, frequency of blood gas sampling is reported in Additional file 2: Table S1.

### Pilot sampling and threshold selection

We conducted a pilot review of 100 patients identified by a 24-h physiologic Berlin screen and found that only 6% met the full case definition after expert review, motivating a persistence-based (≥ 72 h) enrichment strategy informed by autopsy data linking diffuse alveolar damage to sustained hypoxaemia [20].

### Cohort identification: 72-h physiologic screen and adjudication (derivation)

In MIMIC-IV, ICU stays were screened for P/F < 300 mm Hg recorded contemporaneously with PEEP ≥ 5 cm H_2_O sustained for ≥ 72 h (or ≥ 48 h with death on day 3). For patients with multiple ICU admissions, the first episode meeting physiologic screening criteria was retained. A random sample of 2000 screen-positive admissions underwent adjudication for ARDS with cases labelled as ARDS, ARDS possible, and non-ARDS acute hypoxaemic respiratory failure (AHRF). The adjudication sample size was chosen to estimate the prevalence of “true” ARDS within the 72-h screen-positive cohort (*N* = 3940) with acceptable precision and was not a power calculation for between-group outcome comparisons. Assuming a conservative prevalence of 50%, review of 2000 cases yields an approximately ± 1.5% margin of error after finite-population correction.

Adjudication followed a standardised workflow integrating the discharge summary, radiology reports, echocardiography reports, and clinical narrative (Additional file 2: Fig. S1). Cardiogenic and hydrostatic causes of pulmonary oedema were not excluded algorithmically prior to chart review. Timing concordance between hypoxaemia and ancillary criteria was inferred from the clinical narrative. Additional clinical concepts (e.g., direct vs. indirect ARDS, pneumonia, concurrent cardiac failure) were recorded using a prespecified rubric (Additional file 2: Table S2). Primary review was conducted by an experienced clinician reviewer (DCM) with expertise in mechanical ventilation and ARDS. A random subset of 100 charts underwent independent review (MK) to estimate inter-observer agreement; disagreements were resolved by discussion using prespecified rules.

### Radiology report verification

The main case adjudication used radiology reports (chest radiograph and CT). Where chest radiographs were available in MIMIC-CXR we obtained up to three images closest to onset for independent, blinded reads to validate the use of reports as a surrogate. Additional details are provided in Additional file 2: Methods S1.

### Sensitivity and missed case analysis

To examine robustness of the 72-h threshold, we applied three shorter windows after completing the primary analysis: (1) Anytime: criteria met on at least one occasion during the ICU stay; (2) 24-h: criteria met for ≥ 24 h; and (3) 48-h: criteria met for ≥ 48 h. From each screen we drew an independent random sample of 100 admissions for adjudication using the identical rubric. No radiology keyword or ICD enrichment was applied to these sensitivity cohorts. In addition, to assess potential exclusions under the 72-h rule, we identified patients meeting criteria for 0–24 h and 24–48 h and reviewed 100 admissions from each group to perform a missed case analysis.

### External validation (ICHT)

For external validation, we identified 100 random ICU admissions in the ICHT dataset meeting the 72-h screen and performed chart and radiology review as above to adjudicate ARDS prevalence.

### Alternative enrichment strategies (applied within the 72-h cohort)

We evaluated two common retrospective enrichment approaches within the 72-h cohort: (1) radiology-report keyword rules and (2) International Classification of Disease (ICD) 9/10 diagnosis codes. Six Boolean rule-sets (T1–T6; Additional file 2: Table S3) derived from prior work were executed against radiology-report text within two windows: 0–48 h relative to onset and any time during admission; labels were compared with expert adjudication. For ICD benchmarking, we retrieved ARDS-related codes including J80 (ARDS) and broader respiratory-failure codes (Additional file 2: Table S4). Diagnostic accuracy metrics were computed against the adjudicated ARDS label.

### Statistical analysis

Inter-observer agreement was assessed with Cohen’s *κ*, with expected agreement calculated under three categories (ARDS, possible ARDS, and non-ARDS AHRF). Descriptive statistics used medians with interquartile ranges for continuous variables and frequencies with percentages for categorical variables. Wilson intervals were used for binomial proportions. We examined whether the ARDS label was associated with ICU mortality initially with univariate logistic regression followed by multivariate adjustments. Modelling details are described in Additional file 2: Methods S2. Analyses were conducted in R (version 4.3.3); Structured Query Language (SQL) was executed on Google Cloud Platform.

### Reporting guideline

This report conforms to STROBE (Strengthening the Reporting of Observational Studies in Epidemiology); the completed checklist is provided as Additional file 1.

## Results

### Identification of ARDS cohort using physiological parameters and expert review results

**Fig. 1:**
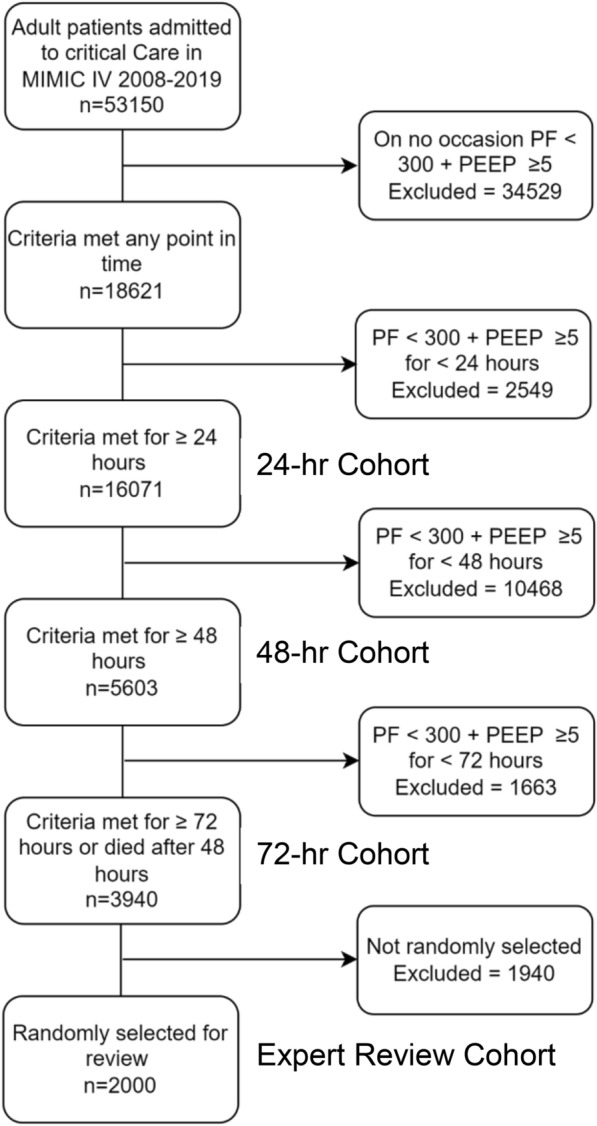
Consort diagram demonstrating increasingly prolonged physiologic criteria (of P/F < 300 + PEEP ≥ 5 cm H2O) for selecting potential subjects with ARDS

Figure 1 illustrates the progressive enrichment of the sample by applying increasingly prolonged physiologic criteria. A total of 18,621 patients (35.0% of the MIMIC-IV cohort) met the criteria of P/F ratio < 300 mm Hg and PEEP ≥ 5 cm H_2_O at any time. Of these, 16,072 patients (30.0%) met the criteria for 24 h, and 5603 patients (10.5%) met the criteria for 48 h. Finally, 3940 patients (7.4%) met the stringent criteria of 72 h with P/F < 300 mm Hg and PEEP ≥ 5 cm H_2_O, or 48 h with these criteria and death on the third day (72-h cohort). In the 72-h cohort 3330/3940 (84.5%) were mechanically ventilated at the point of meeting criteria and 3741/3940 (95.0%) were mechanically ventilated after meeting criteria. 91 subjects died within the first 48 h and by design were excluded. These would have constituted ~ 2% of the final cohort. Descriptive statistics for each progressively enriched cohort are presented in Additional file 2: Table S5.

From the 3940 patients who met the 72-h physiologic screen, we drew a simple random sample of 2000 admissions for expert adjudication. Expert review confirmed ARDS in 993 (49.7%), classified 879 (44.0%) as non-ARDS AHRF and labelled 128 (6.4%) possible ARDS. Characteristics of each labelled group are presented in Table 1.

**Table 1:** Characteristic of expert review cohort—2000 subjects who met all criteria (P/F < 300 mm Hg with PEEP ≥ 5 for 72 h or for 48 h and died) divided into three groups after expert review

Variable	ARDS	ARDS possible	Non-ARDS AHRF
*Count*	993	128	879
*Demographics*			
Male, *n* (%)	622 (62.6)	84 (65.6)	544 (61.9)
Age (IQR)	61 (51–72)	67 (57–75)	66 (57–76)
White, *n* (%)	636 (64.0)	94 (73.4)	578 (65.8)
Black, *n* (%)	72 (7.3)	10 (7.8)	68 (7.7)
Hispanic/Latino, *n* (%)	42 (4.2)	4 (3.1)	31 (3.5)
Asian, *n* (%)	21 (2.1)	1 (0.8)	17 (1.9)
American Indian/Alaska Native, *n* (%)	3 (0.3)	0 (0.0)	1 (0.1)
Race Unknown, *n* (%)	166 (16.7)	12 (9.4)	141 (16.0)
*Comorbidities*			
Ischaemic heart disease, *n* (%)	140 (14.1)	15 (11.7)	226 (25.7)
Cardiac failure, *n* (%)	278 (28.0)	31 (24.2)	356 (40.5)
Stroke, *n* (%)	106 (10.7)	26 (20.3)	173 (19.7)
Chronic kidney disease, *n* (%)	206 (20.7)	28 (21.9)	214 (24.3)
Chronic pulmonary disease, *n* (%)	340 (34.2)	42 (32.8)	273 (31.1)
Diabetes, *n* (%)	288 (29.0)	35 (27.3)	312 (35.5)
Cancer, *n* (%)	150 (15.1)	18 (14.1)	91 (10.4)
Liver disease, *n* (%)	235 (23.7)	23 (18.0)	176 (20.0)
*Admission parameters*			
Direct ARDS, *n* (%)	806 (81.2)	77 (60.2)	NA
Pneumonia, *n* (%)	725 (73.0)	65 (50.8)	14 (1.6)
Pancreatitis, *n* (%)	50 (5.0)	5 (3.9)	2 (0.2)
Trauma, *n* (%)	79 (8.0)	14 (10.9)	37 (4.2)
Surgery, *n* (%)	196 (19.7)	55 (41.9)	368 (43.0)
Onset of acute hypoxaemic respiratory failure, hours (IQR)	8 (2–40)	7 (3–41)	8 (3–39)
*Physiological parameters*			
Mechanical ventilation at onset, *n* (%)	731 (73.6)	91 (71.1)	693 (78.8)
Mechanical ventilation after onset, *n* (%)	980 (98.7)	124 (96.9)	854 (97.2)
Heart rate, bpm (IQR)	90 (78–102)	89 (76–106)	86 (76–98)
Mean arterial pressure, mm Hg (IQR)	74 (69–80)	76 (70–82)	74 (69–81)
Vasopressor use (first 24 h), *n* (%)	383 (38.6)	47 (36.7)	442 (50.3)
Respiratory rate, (IQR)	22 (19–26)	20 (18–24)	20 (18–23)
Tidal volume, ml/kg (IQR)	7.2 (6.4–8.2)	7.1 (6.4–8.2)	7.4 (6.6–8.4)
P/F ratio, mm Hg (IQR)			
*Day 1*	170 (131–215)	189 (157–227)	196 (155–239)
*Day 2*	189 (151–234)	190 (158–234)	205 (165–248)
*Day 3*	196 (157–239)	199 (162–245)	212 (170–255)
PEEP, cm H2O (IQR)			
*Day 1*	10 (7–12)	8 (6–10)	8 (5–10)
*Day 2*	10 (7–12)	10 (7–11)	8 (5–10)
*Day 3*	10 (7–12)	9 (6–11)	8 (5–10)
PaCO2, mm Hg (IQR)	43 (38–49)	41 (37–47)	40 (36- 45)
pH, (IQR)	7.35 (7.29–7.40)	7.36 (7.30–7.41)	7.37 (7.32–7.41)
*Laboratory parameters*			
Sodium, mmol/L (IQR)	139 (136–142)	139 (137–141)	139 (136–142)
Potassium, mmol/L (IQR)	4.1 (3.8–4.6)	4.2 (4.0–4.6)	4.2 (3.9–4.6)
Creatinine, mg/dL (IQR)	1.2 (0.8–1.9)	1.4 (0.8–2.1)	1.3 (0.9–2.1)
Haemoglobin, g/dL (IQR)	10.0 (8.7–11.7)	9.9 (9.0–11.2)	10.0 (9.1–11.6)
Platelets, × 10^9^/L (IQR)	181 (114–256)	175 (123–241)	171 (118–241)
White blood cells, × 10^9^/L (IQR)	12.6 (8.7, 17.7)	12.4 (9.2–17.3)	12.8 (9.6–16.9)
Lactate, mmol/L (IQR)	1.7 (1.2–2.6)	1.9 (1.2–3.3)	2.1 (1.3–3.7)
*Outcomes*			
ICU length of stay, days (IQR)	12.1 (7.9–19.0)	13.3 (8.8–19.4)	9.1 (5.7–14.7)
ICU mortality, *n* (%)	276 (27.8)	28 (21.9)	304 (34.6)
Hospital mortality, *n* (%)	304 (30.6)	38 (29.7)	341 (38.8)

For physiological parameters are reported as median values for the 24-h period after onset of hypoxaemia, laboratory values on the day of onset of hypoxaemia are reported

Inter-observer agreement was 86% (*κ* = 0.76) in a sample of 100 cases; no disagreements occurred between ARDS and non-ARDS AHRF, all were between ARDS versus possible ARDS or possible ARDS versus non-ARDS AHRF (Additional file 2: Table S6). Of the 2000 adjudicated cases, 660 had extractable CXR reports. A subset of 352 cases with linked, readable images nearest to onset underwent blinded image review; report-based labels agreed with image reads in 320/352 (91.0%). Further review of other imaging modalities indicated misclassification in 2/352 (0.6%) cases (Additional file 2: Results S1).

### Sensitivity analysis for time thresholds

When the physiological screen was applied without any persistence requirement (*n* = 100 sample), only 6/100 patients were adjudicated as ARDS (PPV 6%, 95% CI 2–12%). Yield rose modestly to 8% for ≥ 24 h (24-h cohort) and 21% ≥ 48 h (48-h cohort) compared with ~ 50% with the ≥ 72 h criterion (72-h cohort) (Table 2). Inclusion of “possible ARDS” cases increased the respective PPVs to 10%, 10%, 26%, and 57% for any occasion, ≥ 24, ≥ 48, and ≥ 72 h respectively. Because screen-negative patients were not adjudicated, conventional sensitivity and specificity of the persistence screen cannot be estimated. The values reported in Table 2 represent positive predictive value/diagnostic yield within each screen-positive cohort.

**Table 2:** Diagnostic yield of Berlin-physiology persistence windows

Screen window	*n* Reviewed	ARDS	ARDS possible	Non-ARDS AHRF	PPV (95% CI)
*MIMIC-IV*					
Any time (≥ 1 measurement)	100	6	4	90	6 (2–12)
24-h cohort	100	8	2	90	8 (3–14)
48-h cohort	100	21	5	74	21 (13–30)
72-h (Final cohort)	2000	993	128	879	50 (48–52)
*ICHT*					
72-h (Validation cohort)	100	56	5	39	56 (47–66)

For each window: (1) Any-time (≥ 1 qualifying measurement), (2) ≥ 24 h, and (3) ≥ 48 h: 100 randomly selected screen-positive patients underwent expert adjudication and were classified as ARDS, Possible ARDS, or Non-ARDS acute hypoxaemic respiratory failure (AHRF*)*. The positive predictive value (PPV) and its 95% Wilson confidence interval reflect the proportion adjudicated as definite ARDS within each screened sample

We performed a missed case analysis in 100 subjects who were hypoxaemic for 0–24 and 24–48 h to assess whether the 72-h criteria were excluding significant number of subjects. 4/100 and 12/100 of the 0–24 h and 24–48 h cohorts were adjudicated as ARDS respectively.

### External validation

In the ICHT external validation cohort of subjects who met the 72-h criteria, 56/100 were classified as ARDS and 5/100 were classified as possible ARDS.

### Alternative enrichment strategies for identification of ARDS

We assessed whether specific radiological keyword searches applied to chest radiograph reports improved the identification of ARDS cases compared to expert clinical review. Of the 2000 subjects reviewed by experts, 660 had extractable chest radiograph reports available in the MIMIC-CXR database.

We tested six sets of keyword search terms (Additional file 2: Table S3), each progressively broader, aiming to capture radiological features consistent with ARDS within 48 h of hypoxaemic respiratory failure onset (Table 2). Sensitivity ranged from 43% (most narrow terms, T1) to 77% (broadest terms, T6), while specificity decreased from 78% (most specific) to 29% (broadest). Radiology keyword set T3 balanced performance best (sensitivity 49%, specificity 76%).

We also evaluated the utility of ICD codes in ARDS identification. Of the 993 expert-labelled ARDS cases, 756 subjects had at least one ARDS-related ICD code resulting in a sensitivity of 76% and specificity of 47% in the 2000 subject expert review cohort. For the 879 cases labelled as non-ARDS AHRF, 451 (51.3%) had at least one ARDS-related ICD code.

### Characterisation of ARDS versus non-ARDS AHRF cohorts

Descriptive statistics for cohorts defined as ARDS after expert review, possible ARDS and non-ARDS AHRF are reported in Table 1. Most patients classified as ARDS had a direct cause (806/993, 81.2%) and most had pneumonia (725/993, 73.0%). Cardiac failure and surgery were more common in the non-ARDS AHRF group compared to the ARDS group, at 40.2% versus 28.6% and 43.0% versus 19.8%, respectively. In addition, patients in the non-ARDS AHRF group were more frequently in shock, requiring vasopressors, and had higher levels of lactate. In both groups the majority of patients were mechanically ventilated when meeting criteria and almost all subsequently required mechanical ventilation (980/993 [98.7%] and 857/879 [97.2%] for ARDS and non-ARDS AHRF respectively). ECMO support was received by 12 patients (1.2%) in the ARDS cohort and 12 patients (1.4%) in the non-ARDS AHRF cohort.

**Fig. 2:**
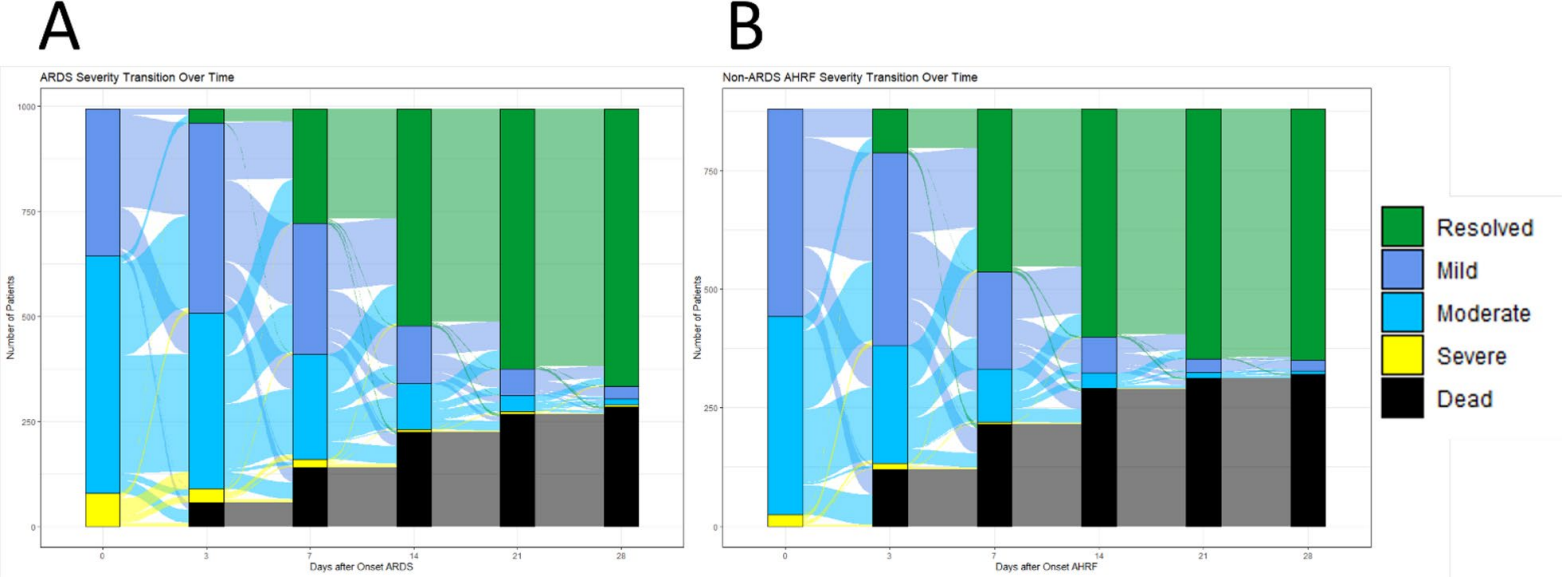
Severity of acute hypoxaemic respiratory failure over time for patients with **A** ARDS and **B** without ARDS. Mild (P/F ratio 200–300 mm Hg), Moderate (P/F ratio 100–200 mm Hg) and Severe (P/F ratio < 100 mm Hg). Time 0 is when subjects first meet P/F and PEEP physiologic criteria

Despite more severe and prolonged hypoxaemia (median P/F ratio at baseline—118 [IQR 86–156] vs. 158 [114–214] and slower resolution over 28 days; Fig. 2), ARDS patients had lower ICU (27.8% vs. 34.6%, *p* < 0.001) and hospital (30.6% vs. 38.8%, *p* < 0.001) mortality than non-ARDS AHRF. They also stayed longer (median ICU length-of-stay 12 days vs. 9 days). Survival curves are shown in Additional file 2: Fig. S2; mortality stratified by Berlin severity is presented in Additional file 2: Table S9.

In univariate analysis ARDS was associated with lower ICU mortality (OR 0.74, 95% CI 0.61–0.90; *p* = 0.002). After adjustment for age, SOFA score, vasopressor dose, comorbidity burden, gender and screened covariates (Additional file 2: Table S7), the association persisted (adjusted OR 0.60, 95% CI 0.42–0.85; *p* = 0.005). The final model showed good fit (AUC 0.76) and excellent calibration (Hosmer–Lemeshow *p* = 0.77; Additional file 2: Figs. S3, S4). Full coefficients and variance inflation factor outputs are provided in Additional file 2: Tables S7, S8.

## Discussion

In a large ICU database with external validation, Berlin physiologic screen (P/F < 300 mm Hg + PEEP ≥ 5 cm H_2_O) with 72-h persistence criterion demonstrated a positive-predictive value (PPV) of 50% in MIMIC-IV and 56% in an external UK cohort. Sensitivity analyses with progressively shorter persistence requirements demonstrated PPV fell steeply as the window shortened: 21% after 48 h of derangement, 8% after a single 24 h period, and only 6% when any isolated measurement met the screen. These gradients confirm that brief or transient hypoxaemia captures a largely heterogeneous AHRF population rather than ARDS. Adding radiology keyword or ICD-code filters did not meaningfully improve discrimination even within the ≥ 72 h enriched cohort.

Our work indicates that previous studies using ‘modified Berlin’ criteria identify a heterogeneous group of patients with AHRF and suggest that persistence ≥ 72 h, not single-time-point screens, more effectively enriches for ARDS. After initial exploratory work we justify the use of persistence for ≥ 72 h for three reasons. First, others have shown that hypoxaemia resolving within 48 h represents a biologically distinct subgroup [21, 22]. Second, diffuse alveolar damage, the histological hallmark of ARDS, is uncommon when hypoxaemia lasts < 72 h, as shown in autopsy series [20]. Third, Madotto et al. separated such patients as “resolved” rather than “confirmed” ARDS [23]. Consistent with that concept, only 8% of our 24-h screen-positives had chart-confirmed ARDS, compared with ~ 50% when derangement persisted ≥ 72 h. Finally, trial cohorts of established ARDS typically remain ventilated for ≥ 7 days [2, 24], so a 3-day requirement is unlikely to exclude clinically meaningful cases yet provides sufficient time for diagnostic imaging and echocardiography to appear in the record.

One-third of ventilated MIMIC-IV patients met Berlin physiology for a 24-h period, yet two-thirds normalised within 48 h. This 65% “fast-resolver” rate dwarfs the 24% seen prospectively in LUNG-SAFE [23], underscoring how retrospective screens inflate apparent ARDS prevalence. Even after a 72 h filter, ~ 4% of all ICU admissions likely had ARDS, implying that many published ‘ARDS’ database cohorts may actually represent heterogeneous AHRF. Although this may be specific to the two databases studied here, it is highly relevant because many studies still apply single-time-point or do not clarify their use of “modified Berlin” filters when developing prediction models or testing interventions.

Text-based enrichment strategies added little discriminatory value. Six radiology keyword rule-sets were evaluated and the best keyword set achieved 49% sensitivity and 76% specificity (overall accuracy 62%), whereas ICD codes reached 76% sensitivity but only 47% specificity. These findings reaffirm prior work showing low sensitivity of ICD-based methods and the limitations of rule-based text searches [25, 26]. Taken together, they emphasise that neither radiology keywords nor diagnosis codes can substitute for expert review or more sophisticated multimodal algorithms when assembling high-fidelity ARDS cohorts.

Recent retrospective studies have operationalised alternative ARDS definitions in MIMIC-IV. Qian et al. applied the new global definition using text-based extraction from radiology reports to identify bilateral infiltrates, reporting ARDS in approximately 18% of ICU admissions; however, no expert adjudication was performed and cardiogenic oedema was not excluded [27]. Our data demonstrate that radiology keyword searches lack both sensitivity and specificity for ARDS even within an already-enriched cohort. Erlebach et al. identified ARDS patients in MIMIC-IV and SICdb using ICD codes alone [28]; our findings and the wider literature confirm that ICD codes for ARDS are characterised by under-coding and poor specificity, with over half of our adjudicated non-ARDS AHRF cases carrying ARDS-related ICD codes. Our adjudicated ARDS prevalence of approximately 3.7% of all ICU admissions is substantially lower than the rates reported in these studies, consistent with the potential for misclassification that physiologic screening without persistence or expert adjudication may introduce. In low-prevalence syndromes such as adjudicated ARDS in retrospective ICU datasets, scale does not compensate for weak phenotype validity. Even modest false-positive rates can significantly bias association studies, phenotyping efforts, and machine-learning models.

There has been significant progress on machine learning detectors which promise scalability and continue to demonstrate improved accuracy [10, 29, 30]. However, the most accurate models were trained on cohorts that clinicians manually curated and often ‘clean’ datasets. These processes are time consuming and challenging to replicate. Further, clinicians at the bedside rarely diagnose ARDS the moment hypoxaemia occurs; they wait to see whether hypoxaemia and imaging changes persist. Most algorithms use no time window or inherit whatever time-window their local experts selected. Previous work has described a “computable ARDS” and urged the development of a shared retrospective definition plus a minimum EHR data set to accelerate model building and external validation [31]. Our results support this: using consensus-driven EHR criteria with an added persistence requirement to reduce transient hypoxaemia. Agreeing on a temporal filter or other methods for enriching a cohort of subjects with AHRF for ARDS would standardise future machine learning work and reduce the manual-review burden.

Non-ARDS AHRF in our cohort carried higher ICU mortality than adjudicated ARDS (34.6% vs. 27.8%), despite lower P/F ratios in the ARDS group. However, non-ARDS AHRF is a heterogeneous residual category rather than a coherent syndrome, meaning these outcome differences likely reflect case-mix rather than an intrinsic property of the comparator group. For example, heart failure, shock, and vasopressor use were all more prevalent in non-ARDS AHRF. Further, survivorship bias introduced by the 72-h rule may contribute to this disparity; patients with rapidly fatal cardiogenic or embolic causes are excluded, while those surviving to 72 h with ongoing multi-organ failure are captured. The prospective LUNG-SAFE study reported a similar pattern, with congestive-heart-failure AHRF exceeding ARDS mortality [32]. Additionally, median tidal volumes in the adjudicated ARDS group (7.2 ml/kg predicted body weight) suggest that ARDS may not have been consistently recognised at the bedside, this is supported by prior prospective data on under-recognition [26]. Alternatively, this observation may highlight that retrospectively adjudicated and clinically recognised ARDS are overlapping but non-identical populations.

Whilst requiring 72-h persistence has advantages, it risks excluding rapidly resolving or fulminant fatal ARDS and may preferentially select treatment-refractory cases. Our missed-case analysis suggests this cost is modest (4% and 12% adjudicated ARDS in the 0–24 h and 24–48 h groups respectively), but because screen-negative patients were not adjudicated, our study estimates PPV rather than sensitivity across the full ICU population. Mild ARDS cases fluctuating near the P/F 300 threshold may be systematically under-represented, and the 72-h rule should therefore be understood as an enrichment strategy for persistent ARDS rather than a comprehensive epidemiologic case definition. An additional limitation of our work is the subjectivity in diagnosing ARDS, particularly in the context of retrospective review of patient records. Exact diagnostic labelling is limited by our reliance on EHR data lacking unrecorded bedside context, as well as the use of a single primary clinician reviewer. Nevertheless, using pre-defined criteria, a 100-case secondary review achieved substantial agreement (86%, *κ* = 0.76) superior to previous ARDS diagnostic literature, with discrepancies limited solely to adjacent possible and confirmed categories [33]. Further, although we performed an external validation the majority of our analysis was performed using the MIMIC-IV database which is a single centre, retrospectively generated research database. Identifying interventions like proning and reliably extracting the temporal relationship between ARDS risk factors and hypoxia onset from structured diagnostic codes is challenging in MIMIC-IV, reflecting a broader limitation of retrospective case-finding.

Physiology-based Berlin screening alone is inadequate for retrospective ARDS identification. A ≥ 72-h persistence rule materially improves enrichment but does not define ARDS; rather, it yields a persistent ARDS-enriched cohort with substantial residual misclassification. These findings have important implications for research using routinely collected data, where reliance on physiology alone risks generating heterogeneous cohorts that do not represent a consistent ARDS population. EHR-based algorithms, including the approach described here, should therefore be interpreted as tools for cohort enrichment and standardisation rather than substitutes for real-time clinical adjudication. A consensus retrospective ARDS definition, pairing the Berlin physiology with a minimum persistence window and basic rule-out data, would standardise case-finding, reduce manual-review burden, and strengthen future machine-learning or epidemiological work in large databases.

## Supplementary Information

Additional file 1.

Additional file 2.

## Data Availability

The MIMIC IV database is publicly available via https://physionet.org/content/mimiciv/3.1/ for researchers who have signed the data user agreement and completed the required training. All code is available on request to the corresponding author.
