# Supplementary material for: Improving retrospective ARDS case-finding using a simple 72-h physiologic persistence rule
Source: Intensive Care Med Exp. 2026 Apr 30;14:58. doi: 10.1186/s40635-026-00901-9 (PMC13133308; doi:10.1186/s40635-026-00901-9)
Supplement: Supplementary file 1 — Additional file 1. [file 40635_2026_901_MOESM1_ESM.docx]

| **#** | **STROBE item** | **Recommendation** | **Page number** | **Manuscript location** |
| --- | --- | --- | --- | --- |
| **Title & Abstract** |  |  |  |  |
| 1 | Title / design | Indicate study design in title or abstract | 1 | Title: “…Retrospective Identification…” + Abstract, first sentence |
| 2 | Abstract | Provide informative, balanced summary | 2 | Structured Abstract (Background, Objectives, Methods, Results, Conclusions) |
| **Introduction** |  |  |  |  |
| 3 | Background/rationale | Explain scientific background and rationale | 3 | Introduction, ¶1–¶4 |
| 4 | Objectives | State prespecified objectives & hypotheses | 3 | Introduction, final paragraph |
| **Methods** |  |  |  |  |
| 5 | Study design | Present key design elements early | 4 | Methods, “Study overview” |
| 6 | Setting | Describe setting, locations, dates | 4 | Methods, “Data sources” |
| 7 | Participants | Eligibility, selection, follow-up | 4 | Methods, “Identification of potential ARDS cohort” + Fig 1 CONSORT-style flow |
| 8 | Variables | Define outcomes, exposures, predictors | 5, S4 | Methods, “Identification…”, “Alternative enrichment strategies”, Statistical analysis |
| 9 | Data sources / measurement | Detail sources & assessment methods | 4 | Methods, “Data source”, “Expert review”, “Radiology-report check” |
| 10 | Bias | Describe efforts to address potential bias | 9-10 | Methods, Sensitivity analyses (24-h, 48-h screens); Discussion (limitations) |
| 11 | Study size | Explain how sample size was arrived at | 5 | Methods, Cohort selection  paragraph (2 000 with ±1.5 % margin) |
| 12 | Quantitative variables | Explain handling of quantitative variables | S4 | Methods, “Identification…” (mean PEEP/PF); Statistical analysis (scaled variables, imputation) |
| 13 | Statistical methods | Describe all statistical methods incl. confounding, sub-groups, sensitivity | 5 | Methods, “Statistical analysis” (kappa, descriptive stats, uni-/multivariable, VIF, AUC, HL test) |
| **Results** |  |  |  |  |
| 14 | Participants | Numbers at each stage + reasons for exclusion | 6 | Results, first subsection + Figure 1 |
| 15 | Descriptive data | Give characteristics, missingness | 6-7 | Results, “Characterisation of ARDS vs non-ARDS AHRF” + Table 2 |
| 16 | Outcome data | Report outcome events/summary measures | 7 | Results, “Outcome of ARDS vs non-ARDS AHRF” |
| 17 | Main results | Unadjusted & adjusted estimates with precision | 6-8 | Results, univariate and adjusted ORs; Supplementary Tables 7–8 |
| 18 | Other analyses | Subgroups, interactions, sensitivity | 7-8 | Results, keyword/ICD enrichment; sensitivity analyses (24-h, 48-h); radiology-image check |
| **Discussion** |  |  |  |  |
| 19 | Key results | Summarise key findings with respect to objectives | 8 | Discussion, opening paragraph |
| 20 | Limitations | Discuss study limitations, bias, imprecision | 9 | Discussion, “Whilst requiring 72-h persistence…”, subsequent paragraphs |
| 21 | Interpretation | Cautious overall interpretation | 8-10 | Discussion, final paragraphs before Conclusion |
| 22 | Generalisability | Discuss external validity | 8-10 | Discussion, single-centre limitation & external validation paragraph |
| **Other information** |  |  |  |  |
| 23 | Funding | Source of funding & role of funders | 11 | “Funding and acknowledgements” |
| 24 | Ethical approval | Ethical/IRB approval | 10 | “Ethics” in Declarations |
| 25 | Data accessibility | Where data/code can be accessed | 10 | “Availability of supporting data” |
