# Supplementary material for: Improving retrospective ARDS case-finding using a simple 72-h physiologic persistence rule
Source: Intensive Care Med Exp. 2026 Apr 30;14:58. doi: 10.1186/s40635-026-00901-9 (PMC13133308; doi:10.1186/s40635-026-00901-9)
Supplement: Supplementary file 2 — Additional file 2. [file 40635_2026_901_MOESM2_ESM.docx]

**Supplementary Materials: Retrospective Identification of ARDS from the Electronic Healthcare Record**

Dominic C Marshall^1,2*^, Brijesh V Patel^1^, Anthony C Gordon^1^, David B Antcliffe^1^, Sonali Parbhoo^3^, Matthieu Komorowski^1^

1. Division of Anaesthetics, Pain Medicine and Intensive care, Department of Surgery and Cancer, Imperial College London, United Kingdom
2. Cleveland Clinic London, London, United Kingdom
3. School of Electrical and Electronic Engineering, Imperial College London, London, United Kingdom

*Corresponding author: dominic.marshall12@imperial.ac.uk

**Index**

**Supplementary Methods**

Supplementary Methods 1 – Sensitivity analysis for radiology reports

Supplementary Methods 2 - Univariate and multivariate modelling

**Supplementary Results**

Supplementary Results 1: Results of sensitivity analysis for radiology report check

**Supplementary Tables**

Supplementary Table 1 – Frequency of arterial blood gas sampling

Supplementary Table 2 – Clinical concepts identified in expert review

Supplementary Table 3 – Boolean rule set for chest radiograph keyword search

Supplementary Table 4 – ICD codes for ARDS identification

Supplementary Table 5 – Baseline characteristics of acute hypoxaemic respiratory failure cohorts (24-hr, 48hr and 72hrs)

Supplementary Table 6: Contingency table for second expert reviewer

Supplementary Table 7: Variable coefficients for multivariate model

Supplementary Table 8: Variance inflation factors for multivariate model

Supplementary Table 9: ICU mortality by severity of hypoxaemia

**Supplementary Figures**

Supplementary Figure 1 – Expert review flow diagram for assessing cases

Supplementary Figure 2 – ROC curve for multivariate model

Supplementary Figure 3 – Calibration plot for multivariate model

Supplementary Figure 4 – Survival analysis for ARDS vs Non-ARDS AHRF

**Supplementary Methods 1**: Sensitivity analysis for use of radiology reports

For the main expert review cohort radiology reports were reviewed in place of chest radiograph images. This approach was chosen because reports were available for all subjects as part of their discharge summary and included computed tomography scans of the thorax in addition to chest radiographs. To ensure the reliability of this approach chest radiographs were reviewed where available for subjects (only a subset of subjects in MIMIC-IV database feature in the MIMIC-CXR database). Three radiographs were reviewed for each patient selected on closest time to onset of hypoxaemia prioritising radiographs in the 24-hour period after onset. These were combined with patients labelled as non-ARDS AHRF, or possible ARDS, and the reviewer was blinded to the assignment and clinical information when reviewing the radiograph. Radiograph were classified into four groups:

1. Compatible with ARDS – significant apparent bilateral infiltrates (not effusion or atelectasis)
2. Possible ARDS – bilateral infiltrates which were suspicious for major atelectasis or effusion
3. Not compatible with ARDS – no evidence of bilateral infiltrates
4. Unclassifiable – technical quality of CXR too poor to for classification

**Supplementary methods 2:** Univariate and multivariate modelling

We examined whether the ARDS label was associated with ICU mortality initially with univariate logistic regression followed by multivariate adjustments. The adjusted model forced six a-priori confounders: ARDS label, age, SOFA score, norepinephrine-equivalent dose (µg kg⁻¹ min⁻¹), Charlson Comorbidity Index, sex, and added selected candidate variables that showed *p* < 0.10 in its own bivariable model. Collinearity was addressed by removing any variable with a variance inflation factor (VIF) > 3 . Mean 24-hr lactate was missing in 12 % of cases; we therefore created five imputed datasets using multivariate imputation by chained equations (predictive-mean matching). Models were fitted in each set and pooled with Rubin’s rules to obtain odds ratios (OR) and 95 % confidence intervals (CI). Discrimination (AUC), variance inflation factor, and calibration (Hosmer–Lemeshow test plus decile plot) were also assessed. Analyses were performed using R version 4.3.3.

**Supplementary Results 1:** Results of sensitivity analysis for radiology reports

A sensitivity analysis to validate the accuracy of our method using radiology reports was performed by reviewing 1056 chest radiograph studies for 352 subjects classified as ARDS who had available images in the MIMIC-CXR database. 320/352 (91 %) of subjects classified as ARDS through expert review had at least one chest radiograph considered compatible with ARDS and 335/352 (95 %) had at least one chest radiograph either classified as compatible with ARDS or possibly compatible. For the 17 subjects which did not have compatible or possibly compatible chest radiograph imaging a re-review of all clinical information, including radiology reports, was performed with clear evidence available for ARDS radiological changes in 15/17 patients (see details in table below).

| **Case** | **Imaging reports / clinical narrative** | **Consistent with ARDS** |
| --- | --- | --- |
| 1 | CT imaging reports indicate multifocal pneumonia. | Yes |
| 2 | Clinical team diagnosed with ARDS and required PEEP of 18 to oxygenate, imaging only refers to very low lung volumes. | Yes |
| 3 | CXR imaging reports indicate bibasal consolidation and CT abdomen and pelvis report indicates probable infectious aetiology. | Yes |
| 4 | CT imaging reports indicate bilateral patchy airspace opacities with ground-glass interstitial thickening, most likely represent multifocal pneumonia. | Yes |
| 5 | CT imaging reports indicate bilateral upper and lower lobe atelectasis which subsequent CXR report describes as worsening consolidation. | Yes |
| 6 | CT imaging reports indicate consolidation within both lower lobes, the right middle lobe are in the lingular segment of the left upper lobe, likely representing bilateral pneumonia possibly as a result of aspiration. | Yes |
| 7 | 5 separate chest radiograph reports describe concurrent right upper lobe pneumonia and left lower lobe pneumonia. | Yes |
| 8 | Initial imaging describes bilateral pulmonary contusions as a result of trauma. | Yes |
| 9 | Serial chest radiograph reports severe heterogeneous consolidation throughout the left lung right mid and lower lung , consistent with widespread pneumonia or pulmonary hemorrhage. Clinical narrative describes right sided pneumonia which spread to the left lung. | Yes |
| 10 | CT imaging reports indicate scattered air wall thickening and mucus plugging of the small airways of the lower lobes. Clinical narrative of bacterial pneumonia superadded to influenza pneumonia. | Yes |
| 11 | CT imaging reports indicate right lower lobe and lingula opacities which consistent with aspiration pneumonia or pneumonia. | Yes |
| 12 | CT imaging reports indicate near complete collapse of the right lower lobe. Multiple areas of consolidation and ground-glass change in both lungs which likely represent pulmonary contusions with possible super added infection. | Yes |
| 13 | CT imaging reports indicate bilateral pulmonary contusions and subsequent pneumonia | Yes |
| 14 | Multiple chest radiograph reports indicate evolution of bilateral aspiration pneumonia. | Yes |
| 15 | Serial chest radiograph reports and CT imaging report indicate predominantly left lower lobe collapse with trivial right sided changes in contact of ventilator associated pneumonia. | No |
| 16 | CT imaging reports indicate multifocal pneumonia predominantly on the right side. | No |
| 17 | CT imaging reports indicate bilateral lower lobe consolidation. | Yes |

**Supplementary table 1**: Descriptive statistics for frequency of arterial blood gas sampling and recording of ventilator settings by subject in the MIMIC-IV database for ICU days 1-3.

|  | Mean number of observations | Standard deviation in number of observations | Median number of observations | Interquartile range in number of observations |
| --- | --- | --- | --- | --- |
| Arterial Blood Gas |  |  |  |  |
| ICU Day 1 | 2.5 | 1.7 | 2 | 2 |
| ICU Day 2 | 3.0 | 2.3 | 2 | 3 |
| ICU Day 3 | 2.9 | 2.1 | 2 | 3 |
| Ventilator Setting |  |  |  |  |
| ICU Day 1 | 4.6 | 4.9 | 4 | 4 |
| ICU Day 2 | 6.4 | 7.2 | 6 | 4 |
| ICU Day 3 | 6.6 | 8.5 | 7 | 4 |

**Supplementary table 2 - Clinical concepts identified**

| Cardiac Failure | Cardiac failure e.g. acute exacerbation of atrial fibrillation, significant valvular disease, myocardial stunning after surgery without other evidence of ARDS |
| --- | --- |
| Concurrent_cardiac_failure | In patients with ARDS but with evidence of cardiac failure e.g. reduced ejection fraction / valve failure. |
| Direct-ards | Pneumonia (bacterial, viral, fungal)  Aspiration  Period of mechanical ventilation with description of VILI  Lung contusion e.g. after trauma  Inhalational injury / drowning  Fat emboli, reperfusion injury |
| Indirect_ards | Sepsis without initial pulmonary involvement  Non-thoracic trauma  Transfusion e.g. TRALI  Cardiopulmonary bypass (note – difficult to separate from myocardial stunning)  Pancreatitis  Drug OD  Burns |
| Pneumonia | Clear description of pneumonia – consolidation + microbiology or clinical dx |
| Liver | Acute liver failure as primary cause of ICU admission |
| Renal | Acute renal failure as a primary cause of ICU admission |
| Neuro | Neurological injury as a primary cause of ICU admission |
| Surgery | Binary - did the patient have surgery |
| Pancreatitis | Pancreatitis described in ICU admission |
| Trauma | Trauma involved in patients’ admission |
| Comfort_measures | Patient made comfort measure only during ICU admission |
| ECMO | Patient transferred for ECMO |
| Pregnant | Patient pregnant during ARDS |

**Supplementary Table 3A**: Boolean rule-sets for chest radiograph report keyword search

| **Rule set** | **Boolean expression (SQL LIKE patterns)** |
| --- | --- |
| **T1** | %bilateral% **AND** (%infiltrate% **OR** %opacit%) |
| **T2** | (%bilateral% **OR** (%left% **AND** %right%)) **AND** (%infiltrate% **OR** %opacit%) |
| **T3** | %bilateral% **AND** (%infiltrate% **OR** %opacit% **OR** %consolidation%) |
| **T4** | (%bilateral% **AND** (%infiltrate% **OR** %opacit%)) **OR** %edema% |
| **T5** | (%bilateral% **OR** (%left% **AND** %right%)) **AND** (%infiltrate% **OR** %opacit% **OR** %consolidation%) |
| **T6** | ((%bilateral% **OR** (%left% **AND** %right%)) **AND** (%infiltrate% **OR** %opacit% **OR** %consolidation%)) **OR** %edema% |

**Supplementary table 3B:** Different keyword terms used to identify chest radiograph reports for patients in reviewed ARDS cohort. Values if relevant chest radiograph within 48 hours of onset of physiological criteria or in brackets for on any occasion.

| Search terms | Sensitivity | Specificity | PPV | Accuracy |
| --- | --- | --- | --- | --- |
| T1 | 0.43 (0.77) | 0.78 (0.39) | 0.69 (0.58) | 0.60 (0.59) |
| T2 | 0.60 (0.91) | 0.56 (0.17) | 0.60 (0.55) | 0.58 (0.56) |
| T3 | 0.49 (0.83) | 0.76 (0.3) | 0.69 (0.57) | 0.62 (0.58) |
| T4 | 0.71 (0.95) | 0.35 (0.06) | 0.55 (0.53) | 0.54 (0.53) |
| T5 | 0.66 (0.94) | 0.49 (0.11) | 0.59 (0.54) | 0.58 (0.54) |
| T6 | 0.77 (0.97) | 0.29 (0.02) | 0.55 (0.52) | 0.54 (0.52) |

**Supplementary Table 4 -** International classification of disease codes used to assess for utility in identification of ARDS**:**

| ICD-9 | | ICD-10 | |
| --- | --- | --- | --- |
| 51881 | Acute Respiratory Failure | J80 | Acute respiratory distress syndrome |
| 51882 | Other pulmonary insufficiency, not elsewhere classified | J96 | Respiratory failure, not elsewhere classified |
|  |  | J960 | Acute respiratory failure |
|  |  | J9600 | Acute respiratory failure, unspecified whether with hypoxia or hypercapnia |
|  |  | J9601 | Acute respiratory failure with hypoxia |

**Supplementary Table 5** - Characteristics of cohorts with increasing minimum duration of acute hypoxaemic respiratory failure. 24-hr cohort: P/F <300 with PEEP ≥5 for 24 hours. 48-hr cohort: P/F <300 with PEEP ≥5 for 48 hours. 72-hr cohort: P/F <300 with PEEP ≥5 for 72 hours or for 48 hours and died in the period 48-72 hours. For physiological parameters are reported as median values for the 24-hour period after onset of hypoxaemia, laboratory values on the day of onset of hypoxaemia are reported.

| Variable | 24-hrcohort | 48-hr cohort | Final cohort (72 hour) |
| --- | --- | --- | --- |
| *Count* | 16071 | 5603 | 3940 |
| **Demographics** |  |  |  |
| Male, *n* (%) | 10192 (63.4) | 3385 (60.4) | 2409 (61.1) |
| Age (IQR) | 67 (57-76) | 65 (54-75) | 64 (53-74) |
| White, *n* (%) | 10733 (66.8) | 3604 (64.3) | 2526 (64.1) |
| Black, *n* (%) | 1137 (7.1) | 432 (7.7) | 304 (7.7) |
| Hispanic/Latino, *n* (%) | 516 (3.2) | 198 (3.5) | 138 (3.5) |
| Asian, *n* (%) | 359 (2.2) | 130 (2.3) | 74 (1.9) |
| Indian Alaskan, *n* (%) | 28 (0.2) | 12 (0.2) | 9 (0.2) |
| Race Other, *n* (%) | 774 (4.8) | 252 (4.5) | 189 (4.8) |
| Race Unknown, *n* (%) | 2524 (15.7) | 975 (17.4) | 700 (17.8) |
| **Comorbidities** |  |  |  |
| Ischaemic heart disease, *n* (%) | 3538 (22.0) | 1105 (19.7) | 764 (19.4) |
| Cardiac failure, *n* (%) | 4959 (30.9) | 1919 (34.2) | 1319 (33.5) |
| Stroke, *n* (%) | 2232 (13.9) | 896 (16.0) | 618 (15.7) |
| Chronic Kidney Disease, *n* (%) | 3133 (19.5) | 1261 (22.5) | 867 (22.0) |
| Chronic pulmonary disease, *n* (%) | 4491 (27.9) | 1789 (31.9) | 1247 (31.6) |
| Diabetes, *n* (%) | 5181 (32.2) | 1776 (31.7) | 1256 (31.9) |
| Cancer, *n* (%) | 1790 (11.1) | 728 (13.0) | 515 (13.1) |
| Liver disease, *n* (%) | 2234 (13.9) | 1183 (21.1) | 875 (22.2) |
| **Physiological Parameters** |  |  |  |
| Heart rate, bpm (IQR) | 84 (76-95) | 87 (76-100) | 88 (77-100) |
| Mean arterial pressure, mmHg (IQR) | 74 (69-79) | 74 (69-81) | 74 (69-81) |
| Vasopressor use (first 24hr), *n* (%) | 6928 (43.1) | 2468 (44.0) | 1752 (44.5) |
| Respiratory rate, (IQR) | 19.27 (17-22) | 21 (18-24) | 21 (18-24) |
| Tidal volume, ml/kg (IQR) | 7.3 (6.5-8.4) | 7.3 (6.5-8.4) | 7.2 (6.4-8.3) |
| P/F ratio, mmHg (IQR) | 209.88 (167-251) | 192 (152-238) | 186 (146-232) |
| PEEP, cmH2O (IQR) | 5. (5-8) | 8 (5-10) | 8 (6-11) |
| PCO2, mmHg (IQR) | 41 (37-45) | 41 (36-46) | 41 (36- 47) |
| pH, (IQR) | 7.36 (7.33-7.40) | 7.36 (7.31-7.41) | 7.36 (7.31-7.41) |
| **Laboratory parameters** |  |  |  |
| Sodium, mmol/L (IQR) | 138 (137-141) | 139 (136-142) | 139 (136-142) |
| Potassium, mmol/L (IQR) | 4.3 (4.0-4.6) | 4.2 (3.9-4.6) | 4.2 (3.9-4.6) |
| Creatinine, mmol/L (IQR) | 1.0 (0.8-1.5) | 1.2 (0.8-1.9) | 1.2 (0.80-2.0) |
| Haemoglobin, mmol/L (IQR) | 10.3 (9.2-11.7) | 10.0 (8.9-11.6) | 10.0 (8.9-11.6) |
| Platelets, mmol/L (IQR) | 170 (127-230) | 178 (119- 247) | 177 (117-247) |
| White blood cells, mmol/L (IQR) | 12.4 (9.3-16.2) | 12.5 (9.1-17.1) | 12.6 (9.1-17.2) |
| Lactate (Mean first 24hr), mmol/L (IQR) | 1.8 (1.3-2.8) | 1.9 (1.3-3.1) | 1.9 (1.3-3.2) |
| **Outcomes** |  |  |  |
| ICU Length of Stay, Days (IQR) | 3.7 (1.7- 8.3) | 9.2 (5.5-15.2) | 11.0 (6.9-17.4) |
| ICU mortality, *n* (%) | 2825 (17.6) | 1549 (27.6) | 1271 (32.3) |
| Hospital mortality, *n* (%) | 3304 (20.6) | 1789 (31.9) | 1429 (36.3) |

**Supplementary Table 6:** Contingency table of secondary reviewer (MK) on 100 random cases previously reviewed by primary reviewer (DCM)

|  | | DCM | | |
| --- | --- | --- | --- | --- |
|  |  | Non-ARDS AHRF | ARDS | ARDS Possible |
| MK | Non-ARDS AHRF | 42 | 0 | 0 |
|  | ARDS | 0 | 40 | 4 |
|  | ARDS Possible | 6 | 4 | 4 |

**Supplementary Table 7:** Pooled multivariable logistic-regression coefficients for the association between the ARDS label and ICU mortality (five imputed data sets; odds ratios are exponentiated). All prespecified confounders were retained; additional covariates with bivariable *p* < 0.10 were included. OR < 1 indicates lower odds of death.

| **Predictor** | **Level / Unit** | **Adjusted OR** | **95 % CI** | ***p*-value** |
| --- | --- | --- | --- | --- |
| **ARDS status** *(ref = No-ARDS)* | No-ARDS | — | — | — |
|  | ARDS | **0.6** | 0.42 – 0.85 | 0.005 |
| **Age** (per year) | — | **1.02** | 1.01 – 1.03 | < 0.001 |
| **SOFA on day 1** (per point) | — | **1.12** | 1.08 – 1.17 | < 0.001 |
| **Norepinephrine-equivalent** | — | 1.01 | 0.96 – 1.08 | 0.6 |
| (µg kg⁻¹ min⁻¹, per unit) |  |  |  |  |
| **Charlson Comorbidity Index** (per point) | — | **1.11** | 1.05 – 1.16 | < 0.001 |
| **Sex** *(ref = Female)* | Female | — | — | — |
|  | Male | 0.98 | 0.78 – 1.25 | 0.9 |
| **Pancreatitis** | Absent | — | — | — |
|  | Present | 0.59 | 0.25 – 1.24 | 0.2 |
| **Pneumonia** | Absent | — | — | — |
|  | Present | 1.13 | 0.79 – 1.62 | 0.5 |
| **Recent surgery** | No | — | — | — |
|  | Yes | **0.25** | 0.19 – 0.33 | < 0.001 |
| **Trauma** | No | — | — | — |
|  | Yes | 0.56 | 0.28 – 1.03 | 0.074 |
| **Lactate** (mmol L, per unit) | — | **1.13** | 1.06 – 1.21 | < 0.001 |
| **PaO₂/FiO₂ 0–24 h** (per 10 mm Hg)† | — | 1 | 1.00 – 1.00 | 0.017 |
| **Serum bicarbonate** (mmol L, per unit) | — | 0.97 | 0.95 – 1.00 | 0.036 |

† Coefficients for PaO₂/FiO₂ are scaled per 10 mm Hg to aid interpretability.

**Supplementary Table 8:** Variance-inflation factors (VIF) for predictors in the final multivariable model. All VIF values are < 3, indicating no meaningful multicollinearity among the retained covariates.

| **Predictor** | **VIF** |
| --- | --- |
| ARDS label | 2.47 |
| Age (per yr) | 1.46 |
| SOFA day 1 | 1.30 |
| Norepinephrine-equivalent | 1.19 |
| Charlson Index | 1.44 |
| Sex (M vs F) | 1.01 |
| Pancreatitis | 1.05 |
| Pneumonia | 2.34 |
| Recent surgery | 1.11 |
| Trauma | 1.04 |
| Lactate (mmol L⁻¹) | 1.37 |
| PaO₂/FiO₂ 0–24 h (per 10 mm Hg) | 1.07 |
| Bicarbonate (mmol L⁻¹) | 1.27 |

**Supplementary Table 9:** ICU mortality by severity of hypoxaemia – mean P/F ratio mmHg for first 24 hours after onset of hypoxaemia.

| P/F Ratio, mmHg | <100 | | 100–200 | | 200–300 | |
| --- | --- | --- | --- | --- | --- | --- |
| Classification | ARDS | Non-ARDS AHRF | ARDS | Non-ARDS AHRF | ARDS | Non-ARDS AHRF |
| n | 86 | 24 | 567 | 439 | 340 | 416 |
| ICU Mortality, n (%) | 28(32.6%) | 8(33.3%) | 171(30.2%) | 160(36.4%) | 77(22.6%) | 136(32.7%) |


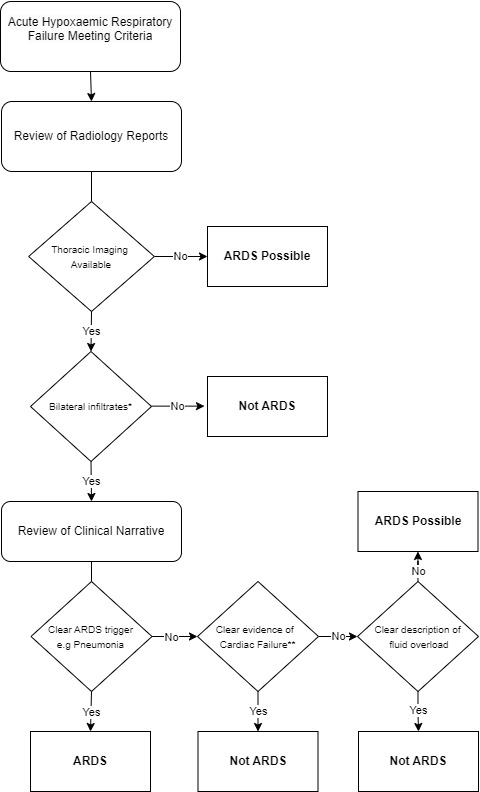


**Supplementary Figure 1**. Flow diagram of expert review of patients records for classifying as ARDS, ARDS possible or not ARDS. *Not simple effusions or minor bibasal atelectasis. ** Systolic or diastolic dysfunction / valve failure on echocardiogram or description of a clinical impression of acute heart failure in clinical narrative.


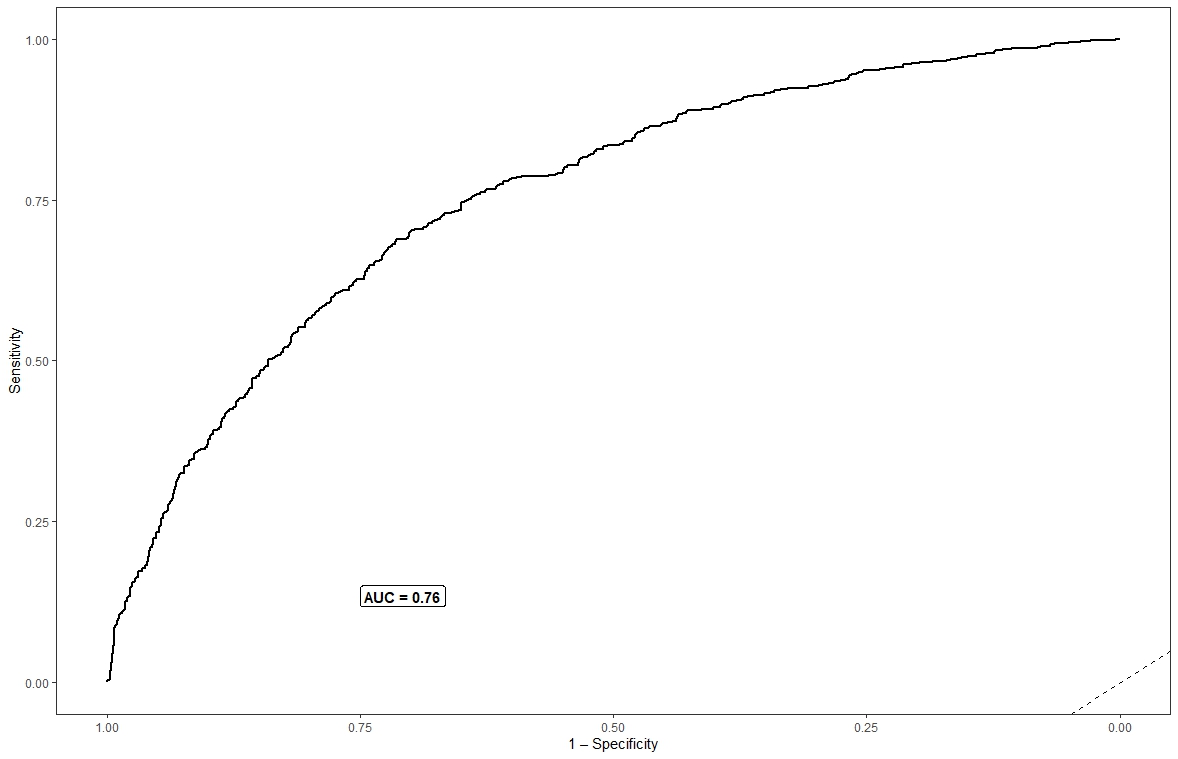


**Supplementary Figure 2:** Receiver-operating-characteristic curve for the multivariable model evaluating the association between the ARDS label and ICU mortality. Predicted probabilities were averaged across the five imputed data sets; the area under the curve (AUC) is 0.76.


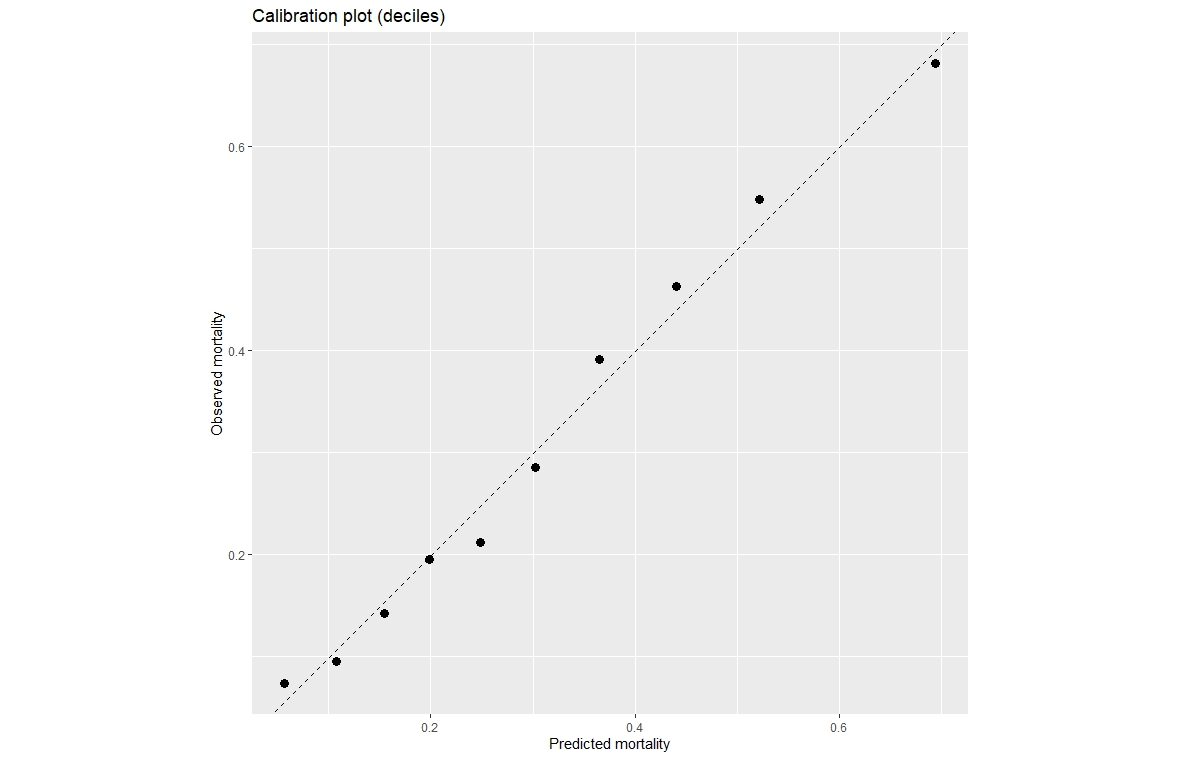


**Supplementary Figure 3:** Calibration plot for the multivariable model shown in Supplementary Figure 2. Observed versus expected ICU-mortality is displayed across risk


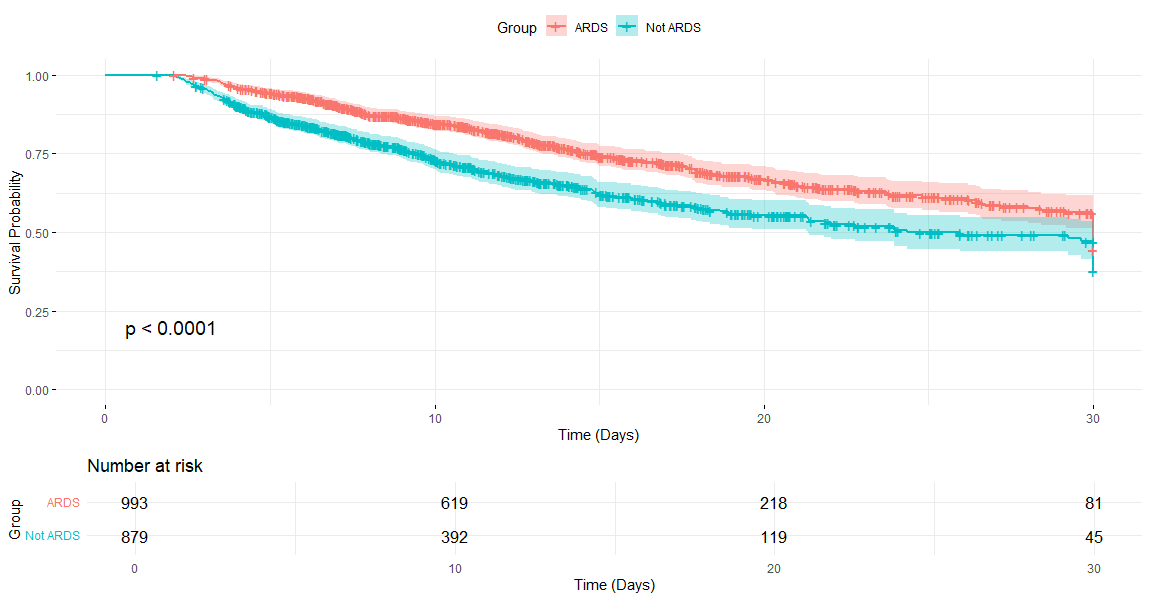


**Supplementary Figure 4:** Survival analysis comparing subjects classified as ARDS vs Non-ARDS AHRF
